# ESPO AND BEHAVIORAL AND SOCIAL SCIENCES SECTION SYMPOSIUM: TAILORING INTERVENTIONS TO REACH AND MEET THE DIVERSE NEEDS OF DIVERSE CAREGIVERS

**DOI:** 10.1093/geroni/igac059.321

**Published:** 2022-12-20

**Authors:** Kylie Meyer, Sara Hackett

## Abstract

Family caregiving cuts across populations, making caregivers a highly diverse population in terms of culture, family organization, care situations, and more. A 2021 report from the National Academies of Sciences Engineering and Medicine reinforces the need to develop and test tailored interventions in order to successfully reach and support family caregivers. Yet, there is limited practical guidance to help researchers to develop intervention programs tailored to the diverse needs of family caregivers. This symposium endeavors to address this gap by sharing accounts from researchers who have effectively tailored existing interventions to meet the diverse needs of diverse caregivers, as well as those who collaboratively worked alongside family caregivers in order to build a tailored program from the ground up. To begin, Kristin Cloyes, PhD MN RN will describe a study where she examines alignment between LGTBQ+ hospice family caregivers and other members of the hospice care teams, as it relates to support and communication needs. Next, Jung-Ah Lee, PhD, RN, FGSA, FAAN, will describe the experiences of racially and ethnically diverse caregivers who received a community health worker intervention to access resources tailored to their language needs. Jaime Perales-Puchalt, PhD, MPH, will describe the results of CuidaTEXT intervention study, which used tailored Short Messaging Service-based text message to deliver support to Latin family caregivers. Lastly, Ishan Williams, PhD, FGSA, will present on findings from research on how community engagement as well as culturally informed intervention can improve the representation of family caregivers, especially African American caregivers, in caregiving research studies

